# Supplementary material for: Content-rich biological network constructed by mining PubMed abstracts
Source: BMC Bioinformatics. 2004 Oct 8;5:147. doi: 10.1186/1471-2105-5-147 (PMC528731; doi:10.1186/1471-2105-5-147)
Supplement: Additional File 2 — The original results of the above study (non-essential files are deleted to keep the file size under the limit set by BMC bioinformatics). [file 1471-2105-5-147-S2.bz2 › chilibotAdditionalFile2/dip10/78ID7565724E290/html/WAS_NCK1.html]

 


 **WAS** and **NCK1** 
  
Found 20 abstracts in PubMed, retrieved 10.  
 

 What does Google say? 
 PDF only 
| .edu only 

---

**Interactive relationship** (e.g. stimulation, inhibition, etc)

**Neutral relationship**- and another through Nck  [ **NCK1** ]  that promotes the recruitment of WASP  [ **WAS** ] .  Ref: 11739662 J Cell Sci, 2001
- Upon induction of phagocytosis, a large molecular complex, consisting in part of Ena VASP proteins, the Fyn binding SLP 76 associated protein Fyb SLAP, Src homology 2 SH2 domain containing leukocyte protein of 76 kDa SLP 76, Nck  [ **NCK1** ] , and the Wiskott Aldrich syndrome protein  [ **WAS** ]  WASP  [ **WAS** ] ,  **was**  formed.  Ref: 11739662 J Cell Sci, 2001
- Phosphatidylinositol biphosphate PIP2 induced vesicle movement depends on N WASP  [ **WAS** ]  and involves Nck  [ **NCK1** ] , WIP, and Grb2.  Ref: 12147689 J Biol Chem, 2002
- The recruitment and activation of WASP  [ **WAS** ]  are coordinated by tyrosine phosphorylated Src homology 2 domain containing leukocyte protein of 76 kDa, which functions as a scaffold, bringing Nck  [ **NCK1** ]  and WASP  [ **WAS** ]  into proximity with Vav 1 and Cdc42 GTP.  Ref: 12874226 J ImmunolJ Immunol, 2002
- Evidence for a molecular complex consisting of Fyb SLAP, SLP 76, Nck  [ **NCK1** ] , VASP and WASP  [ **WAS** ]  that links the actin cytoskeleton to Fcgamma receptor signalling during phagocytosis.  Ref: 11739662 J Cell Sci, 2001
- This process resulting in the motility of intracellular vesicles at the tips of actin comets involved the recruitment of the Src homology 3 SH3 SH2 adaptor proteins Nck  [ **NCK1** ]  and Grb2 as well as of WASP  [ **WAS** ]  interacting protein WIP.  Ref: 12147689 J Biol Chem, 2002

**Non-interactive relationship** (e.g. studied together, co-existance, homology, etc.)

- Nck  [ **NCK1** ]  stimulated actin nucleation by N WASP  [ **WAS** ] .Arp2 3 complexes is further stimulated by phosphatidylinositol bisphosphate, but not by GTP Cdc42, suggesting that Nck  [ **NCK1** ]  and Cdc42 activate N WASP  [ **WAS** ]  by redundant mechanisms.  Ref: 11340081 J Biol Chem, 2001
- The Nck  [ **NCK1** ]  Src homology SH 2 3 adaptor binds via its SH3 domains to a proline rich region on WASP  [ **WAS** ]  and N WASP  [ **WAS** ]  and has been implicated in recruitment of these proteins to sites of tyrosine phosphorylation.  Ref: 11340081 J Biol Chem, 2001
- Moreover, activated Nck  [ **NCK1** ]  interacted with Cas and WASP  [ **WAS** ]  as determined by co immunoprecipitation, and FAK also bound to Cas.  Ref: 12135674 Exp Hematol, 2002
- We show here that Nck  [ **NCK1** ]  SH3 domains dramatically stimulate the rate of nucleation of actin filaments by purified N WASP  [ **WAS** ]  in the presence of Arp2 3 in vitro.  Ref: 11340081 J Biol Chem, 2001
- Our previous studies have shown that N WASP  [ **WAS** ]  is required for the actin based motility of vaccinia virus and is recruited via Nck  [ **NCK1** ]  and WIP.  Ref: 12007418 Curr Biol, 2002
- Nck  [ **NCK1** ]  is required for the recruitment of both neural Wiskott Aldrich syndrome protein  [ **WAS** ]  N WASP  [ **WAS** ]  and the actin related protein Arp 2 3 complex to the EPEC pedestal, directly linking Tir to the cytoskeleton.  Ref: 11533668 Nat Cell Biol, 2001
- Nck  [ **NCK1** ]  and phosphatidylinositol bisphosphate synergistically activate actin polymerization through the N WASP  [ **WAS** ]  Arp2 3 pathway.  Ref: 11340081 J Biol Chem, 2001
- The Grb2 pathway alone is therefore unable to nucleate actin tails, as its recruitment requires the prior recruitment of N WASP  [ **WAS** ]  by Nck  [ **NCK1** ] .  Ref: 12007418 Curr Biol, 2002
- Here, we show that WASP  [ **WAS** ]  recruitment occurs through binding to the C terminal Src homology 3 domain of Nck  [ **NCK1** ] .  Ref: 12874226 J ImmunolJ Immunol, 2002
- SLP 76 Coordinates Nck  [ **NCK1** ]  Dependent Wiskott Aldrich Syndrome Protein  [ **WAS** ]  Recruitment with Vav 1 Cdc42 Dependent Wiskott Aldrich Syndrome Protein  [ **WAS** ]  Activation at the T Cell APC Contact Site.  Ref: 12874226 J ImmunolJ Immunol, 2002
- CONCLUSIONS These data suggest that WASP  [ **WAS** ] , Cas, Nck  [ **NCK1** ] , and FAK may play a role in SDF 1alpha induced migration of the T cell line, Jurkat.  Ref: 12135674 Exp Hematol, 2002
